# Effects of a tailored rehabilitation treatment in lower limb Soft Tissue Sarcomas reconstruction: a case series

**DOI:** 10.1186/s12984-026-01878-y

**Published:** 2026-01-21

**Authors:** Andrea Demofonti, Beniamino Brunetti, Marco Germanotta, Marco Morelli Coppola, Francesca Falchini, Alice Valeri, Stefania Tenna, Sergio Valeri, Irene Giovanna Aprile

**Affiliations:** 1 IRCCS Fondazione Don Carlo Gnocchi, 50143 Florence, Italy; 2 Operative Research Unit of Plastic-Reconstructive and Aesthetic Surgery, Fondazione Policlinico Universitario Campus Bio-Medico, 00128, Rome, Italy; 3 Operative Research Unit of Plastic-Reconstructive and Aesthetic Surgery, Università Campus Bio-Medico di Roma, 00128, Rome, Italy; 4 Operative Research Unit of Soft-Tissue Sarcomas Surgery Department, Fondazione Policlinico Universitario Campus Bio-Medico, 00128, Rome, Italy

**Keywords:** Human biomechanics, Microsurgical reconstruction procedure, Rehabilitation, Soft Tissue Sarcoma

## Abstract

**Purpose:**

The primary treatment for lower limb Soft Tissue Sarcoma (LL-STS) consists of wide surgical resection followed by the Free Functional Muscle Transfer (FFMT) when restoration of muscular continuity and contractile function is needed. Despite the promising results, this approach led to the onset of neuromotor disabilities, reducing the patients’ sensorimotor capabilities during walking. Nowadays, the role of rehabilitation in neuromuscular recovery after FFMT has not been deeply analyzed. The aim of the study was to evaluate the effect of a customized rehabilitation protocol on walking capabilities of patients with LL-STS who underwent radical resection followed by microsurgery reconstruction using FFMT.

**Methods:**

Three patients after wide surgical resection and microsurgical reconstruction followed a personalized rehabilitation protocol according to the site of the lesion (hamstrings or quadriceps). Their ambulation performance was evaluated at the beginning, at the end of rehabilitation, and at long-term follow-up using an optoelectronic system, surface and invasive electromyography. Simultaneously, a clinical survey on physical limitations, post-operative neuropathic pain, and perceived quality of life was submitted to the patients.

**Results:**

The patients showed progressive improvements in lower limb joint kinematics and spatio-temporal parameters for both limbs. These results were confirmed by the electromyography analysis demonstrating a complete reinnervation of the flap in all cases, with muscle activation patterns close to physiological one. Indeed, the patients developed coordinated activation patterns and compensatory strategies in the hamstrings and quadriceps femoris that supported limb stability and joint control during movement. The clinical scales demonstrated both a reduction in neuropathic pain and an improvement in physical functionalities and perceived quality of life.

**Conclusion:**

The proposed rehabilitation approach was effective in enhancing ambulation performance of patients following FFMT. These results highlight the critical role of rehabilitation in maximizing functional outcomes after complex oncologic-musculoskeletal surgeries.

*Trial registration* ClinicalTrials.gov (ID NCT06282237, Registration date: 2024-02-28)

## Introduction

Soft Tissue Sarcomas (STSs) are a group of rare and different malignant tumors representing 1% of all adult oncological diagnoses [1] with 13500 and 24000 new cases each year in the United States of America and the European Union, respectively [1, 2].

Such low incidence and prevalence are complicated by the STSs biological heterogeneity [3] and their wide anatomical distribution, as these neoplasms may arise in any anatomic site [4, 5] with the lower limbs being the most commonly affected region [6].

Although the STSs therapeutic approach is tailored according to both patient-specific factors and tumor-specific characteristics, the surgical resection is the primary treatment [7], eventually combined with adjuvant therapies [8, 9].The recommended radical resection involves a wide excision to achieve negative margins classified as R0 in accordance with the guidelines of the Union for International Cancer Control [10]. Therefore, the incision could create large soft tissue defects which require additional reconstructive plastic surgery treatments.

In this context, a novel promising microsurgical reconstruction is represented by the Free Functional Muscle Transfer (FFMT). This approach involves the extraction from a donor site of a free muscle or myocutaneous flap with microvascular arterial-venous anastomoses and nerve coaptation and then its positioning and insertion into the site of the sarcoma [11]. Despite the promising results obtained in terms of segmental muscle strength recovery observed in some patients with LL-STS at 12 months after microsurgery reconstruction by FFMT [11], the proposed approach inevitably leads to lower limb sensorimotor impairments [12]. This highlights the huge need for rehabilitation addressing all the patient’s neuromotor disorders [12].

Previously, our research group proposed a specific rehabilitation protocol, tailored to the type of surgery performed, in patients with lower extremity impairments following surgery for STSs, demonstrating a significant improvement in motor performance, daily activity ability, walking and pain management, using clinical measures [13]. To the best of our knowledge, no studies have evaluated the effects of a personalized rehabilitation treatment following surgical excision of a sarcoma and subsequent FFMT, using instrumental measures that objectively assess gait impairment after surgery and the subsequent gait recovery process, including possible walking compensatory strategies.

Therefore, the aim of the study was to evaluate the effect of a customized rehabilitation protocol on the walking capabilities of three patients with LL-STS located in the hamstrings or in the quadriceps treated with radical excision followed by FFMT. Instrumental measures (i.e., an optoelectronic system, surface electromyography sEMG and invasive one iEMG) were adopted to evaluate the patients’ ambulation performance at the beginning ($\textit{T}_\textit{0}$, within three months following surgery), at the end ($\textit{T}_{\textit{1}}$, within six months following surgery) of the rehabilitation, and at a long-term follow-up ($\textit{T}_{\textit{2}}$, at least one year after surgery). In addition, clinical scales and patient-oriented questionnaires were used to assess the abilities in daily life, the post-operative neuropathic pain, and the perceived Quality of Life (QoL).

The paper is organized as follows: Section II describes the proposed rehabilitation treatment, the details of the instrumented and clinical assessment, and the data analysis. Section III reports and discusses the obtained results, and Section IV is dedicated to conclusions and future work.

## Materials and methods

### Study design

The study was conducted in the following two main centers: the Fondazione Policlinico Universitario Campus Bio-Medico in Rome, where the three patients were enrolled and underwent surgery for LL-STS radical excision followed by FFMT; the Centro Santa Maria della Provvidenza of the Fondazione Don Carlo Gnocchi in Rome, where the patients underwent a tailored rehabilitation protocol.

The study was approved by the Ethics Committee of the Fondazione Policlinico Universitario Campus Bio-Medico (Protocol number PAR 77.22 OSS), by the Ethics Committee Lazio 1 (Protocol number 420/CE Lazio 1) and registered on ClinicalTrials.gov (ID NCT06282237). In accordance with the Helsinki Declaration and following amendments, the main aspects of the study were explained to the participants in a comprehensive language and they signed an informed consent.

### Participants’ recruitment

The patients admitted to the Fondazione Policlinico Universitario Campus Bio-Medico in Rome between 2019 and 2024 were screened. The inclusion criteria were: (a) age over 18 years; (b) adult patients affected by primary localized STS, candidates for limb/trunk surgery with wide excision or retroperitoneal resection (including resection of the iliopsoas muscle with possible damage to the femoral nerve) with curative intent; (c) defects larger than 100 cm^2^. The exclusion criteria were: (a) recurrent tumors; (b) metastatic diseases; (c) palliative surgery; (d) amputations.

Three patients with STSs were enrolled in the study, and were treated with radical excision followed by FFMT. The main demographic and clinical characteristics of the patients and the details of the surgical procedure are reported in Table 1.

Patient P1 was a 59-year-old male with a pleomorphic STS located in the left thigh postero-medial compartment. The surgical resection implied the biceps femoris and vastus lateralis removal with subsequent reconstruction using a functional flap from the latissimus dorsi. Patient P2 was a 46-year-old male with a diagnosis of low-grade fibromyxoid STS in the left thigh antero-lateral compartment. The surgery induced the partial removal of the main muscles of the quadriceps femoris (i.e., rectus femoris, sartorius, vastus intermedius, vastus lateralis, and vastus medialis) with a reconstruction using a functional flap exported from the contralateral compartment. Patient P3 was a 75-year-old male with a myxofibrosarcoma in the left thigh antero-medial compartment. During surgery, the rectus femoris and the sartorius were removed, and a functional flap from the contralateral compartment was used for reconstruction. The recipient vessels used in cases of microsurgical reconstruction of the thigh anterior compartment included the lateral circumflex femoral artery and vein, and perforators from the superficial femoral artery. The medial sural vessels were used in posterior thigh compartment reconstruction. The recipient nerves included the motor branch of the vastus lateralis and of the hamstrings for anterior and posterior compartment reconstructions, respectively.

**Table 1:** Demographics, tumor characteristics and surgical procedures of each enrolled patient

	P1	P2	P3
Gender	M	M	M
Age [years]	59	46	75
Heigth [cm]	187	167	176
Mass [kg]	85	75	73
Type of STS	Pleomorphic	Low-grade fibromyxoid	Myxofibrosarcoma
Side	L	L	L
Thigh compartment	Postero-medial	Antero-medial and antero-lateral	Antero-medial
Defect size [cm]	18x11x8	18x10x5	16x10x7
Removed muscles	BF, VL	*RF, SR, VI, VL, VM*	RF, SR
Functional flap donor site	LD	ALT-RF	ALT-VL
Recipient vessels	PF-AVp	SF-AVp	LCF-AV
Walking aid	Crutch	Crutch	Crutch
T0 [days from surgery]	65	91	56
T1 [days from surgery]	105	144	107
T2 [days from surgery]	391	443	510

*ALT* Antero-Lateral Thigh,* L* Left,* LCF-AV* Lateral Circumflex Femoral Artery and Vein,* LD* Latissimus Dorsi,* M* Male,* PF-AVp* Perforating branches of the Profunda Femoris Artery,* RF* Rectus Femoris,* SF-AVp* Superficial Femoral Artery and Perforating Vein,* SR* Sartorius,* VI* Vastus Intermedius,* VL* Vastus Lateralis,* VM* Vastus Medialis. The muscles reported in italic were removed partially and not totally

### Rehabilitation protocol

Following surgery, the patients underwent a rehabilitation protocol at Centro Santa Maria della Provvidenza of the Fondazione Don Carlo Gnocchi in Rome.

The rehabilitation treatment aimed to the recovery of the muscular strength, balance, proprioception and lower limb joints’ Range of Motion (ROM).

In the immediate post-operative period (0–15 days), the patients were limited to wheelchair mobility and engaged in postural training, trunk control exercises without weight-bearing on the operated limb. Verticalization was introduced only after 21 days, reflecting the need to minimize mechanical stress on the transferred muscle during the early healing phase. Donor limb rehabilitation began between 15 and 30 days, with a gradual ROM recovery and the initiation of isometric contractions after 15 days. Similarly, passive mobilization and isometric contractions of the operated limb were delayed until after 15 days and 45–60 days, respectively, to allow the tissue integration and neural adaptation. Ambulation with progressive weight-bearing on the operated limb was typically permitted after 30 days, often requiring external support. The rationale, details, and timing of the rehabilitation protocol are reported in Galluccio et al. [13].

The aforementioned exercises were carried out in two 50-minute sessions per day, six days per week, and it implied both a robotic and conventional treatment.

As for the former, the recover of muscular strength, balance, proprioception, and lower limb joints range of motion was pursued using one or more of the following technologies: (i) the end-effector robot G-EO System (Reha Technology, Olten, CH) characterized by body weight support and two footplates inducing locomotor gait pattern; (ii) the end-effector robot Lambda (Lambda Health System, Yverdon-les-Bains, CH) where the patient is seated in a chair and is secured distally by two footplates supporting single and multi-joint movements in passive, assisted, or active mode; (iii) the auto-adaptive instrumented treadmill Walker View (TecnoBody, Dalmine, IT) supporting the patients’ body weight and adapting the speed according to the patient’s residual motor capabilities; (iv) the stabilometric platform Hunova (Movendo Technology, Genova, IT) designed to train patients’ equilibrium and posture in a sitting or standing position. As for the latter, the motor exercises were performed or assisted by physical therapists, mostly in one-to-one sessions. No additional treatment like electrical stimulation or blood flow restriction was adopted.

### Instrumented assessment

Three gait analysis sessions were performed at the beginning ($\textit{T}_{\textit{0}}$, within three months following surgery), at the end ($\textit{T}_{\textit{1}}$, within six months following surgery) of the rehabilitation and at a long-term follow-up ($\textit{T}_{\textit{2}}$, at least one year after surgery) aiming to evaluate the participant’s ambulation performance and the improvement over time.

Gait analysis was performed with the optoelectronic marker-based system BTS Smart D 500 (BTS Bioengineering, Milan, IT). Before each acquisition, eight cameras were mounted on tripods and geometrically calibrated, and twenty-two photo-reflective spherical (diameter of 10 mm) markers were placed on specific anatomical landmarks of the patient’s body according to the Davis protocol [14]. The kinematic data were collected with a sampling rate of 100 Hz. Eight sEMG sensors (FREEEMG, BTS Bioengineering, Milan, IT) were adopted for monitoring the electrical activity of the following muscles of the patients operated limb: (1) Rectus Femoris (RF, responsible for hip flexion and knee extension), (2) Vastus Lateralis (VL, responsible for hip flexion and knee extension), (3) Vastus Medialis (VM, responsible for hip flexion and knee extension), (4) Biceps Femoris (BF, responsible for hip extension and knee flexion), (5) Semitendinosus (ST, responsible for hip extension and knee flexion), (6) Gluteus Maximus (GM, responsible for hip extension), (7) Tibialis Anterior (TA, responsible for ankle flexion), (8) Gastrocnemius Lateralis (GL, responsible for ankle extension). These muscles were selected since they are the superficial ones most involved during physiological ambulation [15]. The electrodes placement was carried out in accordance with the Surface EMG for the Non-Invasive Assessment of Muscles (SENIAM) guidelines [16]. The myoelectric data were collected with a sampling rate of 1000 Hz. In each session, the patients were asked to perform ten barefoot walking trials at a self-selected speed along a straight path of 8.0 m. Since all patients were assisted by a unilateral crutch during walking at the beginning of the rehabilitation protocol, the same aid was employed during all subsequent instrumented assessment sessions, even when patients were able to walk independently. Although the use of such an assistive device may influence both kinematic and kinetic characteristics of gait [17], the crutch did not compromise the analysis and ensured consistency of the experimental conditions, as it was systematically adopted across all sessions. Consequently, the only differences observed between evaluation sessions can be attributed to improvements in the patients’ lower limb neuromotor function.

In the last evaluation session ($\textit{T}_{\textit{2}}$), following the gait analysis, the patients allowed to rest for 15 min before undergoing needle electromyography assessing the presence of spontaneous and voluntary muscular activity in the functional flap. Such analysis was carried out exclusively in the long-term follow-up session because of the need to account for the physiological time course required for nerve and muscle reinnervation following FFMT. In addition, it was not performed during walking to avoid potential tissue injury, motion-related artefacts, and patient discomfort. Furthermore, gait analysis with sEMG sensors was always conducted before needle electromyography to prevent the onset of eventual injuries and pain that could negatively affect the patients’ ambulation performance. During iEMG signals acquisition, the participant was asked to lie down on a sterilized medical bed, and the skin surface was located and cleaned using an alcohol pad. Then, a neurologist performed the analysis using the Natus UltraPro S100 electromyographic system (Natus Neurology Inc., Middleton, WI, United States) and the TECA Elite disposable concentric needle electrodes with a diameter of 0.30 mm (Natus Neurology Inc., Middleton, WI, United States) implanted in a direction perpendicular to the FFMT, ensuring a maximum number of monitored motor fibers. The iEMG signals were acquired with a sampling rate of 48 kHz.

### Clinical assessment

In addition to the instrumental assessment, a clinical evaluation of abilities during daily life, the post-operative neuropathic pain, and the QoL was performed during each session.

The clinical survey was composed of the following evaluation scales and questionnaires:

- The Leeds Assessment of Neuropathic Symptoms and Signs (LANSS) for evaluating the post-operative neuropathic pain [18] since it was already adopted for patients with STS [19]. It is a clinical tool used to distinguish neuropathic pain from non-neuropathic (i.e., nociceptive) one. The scale combines subjective questions regarding the type of symptoms (e.g., burning sensations, tingling, electric shocks) and objective sensory tests (e.g., changes in sensitivity to touch or pinprick). The total score is between 0 and 24, and a score greater than 12 suggests a significant neuropathic component to the pain;
- The Musculoskeletal Tumor Society Rating Scale differentiated for the Lower Limb (MSTS-LL) [20] for evaluating the patients’ pain and activity limitation since it was validated to assess limb function in oncologic patients after surgical treatment for musculoskeletal tumors (e.g., STS) [21]. The scale encompasses six domains, yielding a total score ranging from 0% to 100% with higher scores indicating better functional status. MSTS-LL score between 80% and 100% indicates an excellent function, between 60%-79% a good function, between 40%-59% a moderate function and less than 40% a severely limited function;
- The Toronto Extremity Salvage Score differentiated for the Lower Limb (TESS-LL) [22] for evaluating the physical functionality in daily life perceived by patients since it was validated for those who have undergone surgical treatments for musculoskeletal tumors of the limbs (e.g., STS) [23]. It contains 30 questions, and each of them assesses how difficult it is for the patient to perform daily activities. Each activity is rated on a scale from 0 (impossible to perform) to 5 (no difficulty). The final score ranges from 0% to 100%, with higher scores indicating better function. TESS-LL score between 80% and 100% indicates an excellent function, between 60%-79% a good function, between 40%-59% a moderate function, and less than 40% a severely limited function;
- The Short Form Health Survey 36 (SF-36) [24] for QoL assessment since it is one of the most widely adopted tool in the field [25]. It comprises 36 items evaluating eight health domains (i.e., physical functioning, role functioning, bodily pain, general health, vitality, social functioning, role emotional and mental health) with a score ranging from 0 to 100. Higher results indicate a better QoL;
- The European Organization for Research and Treatment of Cancer Quality-of-Life Questionnaire (EORTC QLQ-C30) for QoL evaluation [26]. It is a cancer-specific, patient reported health-related quality of life instrument that has been validated showing reliable measures [27]. The scale focuses on five functional domains (i.e., physical, role, emotional, cognitive, and social), QoL and eight symptom-related dimensions (i.e., fatigue, nausea and vomiting, pain, dyspnea, insomnia, loss of appetite, constipation, diarrhea, and financial difficulties) with each item scored on a scale from 0 to 100. Higher scores in the functional domains and QoL indicate better functioning and well-being, whereas higher scores in the symptom scales reflect a greater symptom burden.

**Fig. 1:**
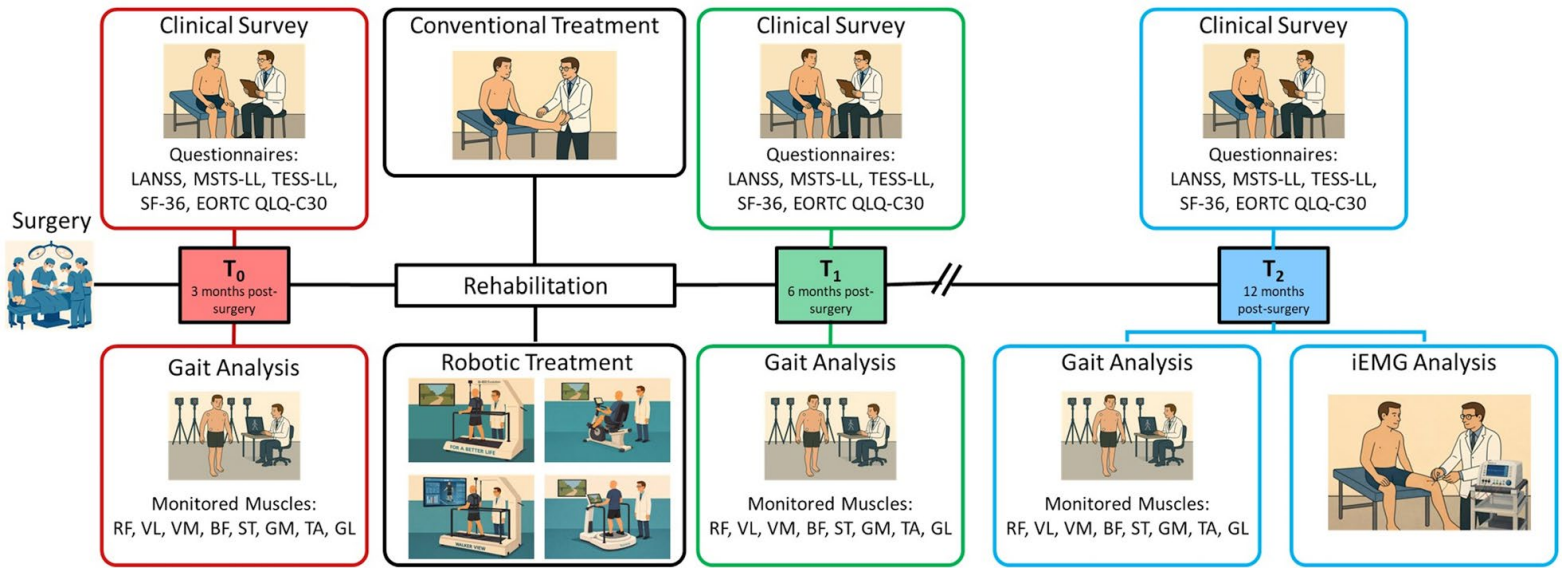
Overview of the study. Following surgery, the patients underwent a 3 months-rehabilitation including both a robotic and conventional treatment. The clinical survey and gait analysis were performed at the beginning ($\textit{T}_{\textit{0}}$, within three months following surgery), at the end ($\textit{T}_{\textit{1}}$, within six months following surgery) of the rehabilitation and at a long-term follow-up ($\textit{T}_{\textit{2}}$, at least one year after surgery). In addition, at $\textit{T}_{\textit{2}}$, an iEMG analysis was executed

An overview of the study detailing the rehabilitation treatment characteristics, the timing and main features of the instrumented assessments are reported in Fig. 1.

### Data analysis and evaluation metrics

The recorded retro-reflective markers were first labeled using a frame-by-frame tracking system (Smart Tracker, BTS Bioengineering, Milan, IT) and then their positions were tracked and reconstructed. Subsequently, the lower limb joint kinematics, the spatio-temporal parameters and the raw sEMG signals were extracted as text files. The iEMG signals were extracted by the proprietary software of the adopted needle electromyography.

The kinematic and sEMG signals, the spatio-temporal parameters and the results of the clinical survey were processed offline in MATLAB (R2022b, MathWorks, Natick, MA, USA).

The sEMG signals acquired during gait were pre-processed by using a second-order Butterworth bandpass filter with cut-off frequencies (10,400) Hz and a second-order Butterworth notch filter (50 Hz) to remove noise from power lines [28]. Subsequently, the signals of each muscle were rectified, enveloped, and then normalized with respect to the minimum and maximum value of myoelectric activity reached by that specific muscle during all the repetitions.

The following indicators were extracted to evaluate the patients’ ambulation performance and clinical status:

- Lower limb joint kinematics: the hip, knee and ankle flexion/extension and the related ROMs (hFE and hROM, kFE and kROM, aFE and aROM, respectively) were computed for both sides to evaluate the kinematic configuration assumed by the participants during walking;
- Spatio-temporal parameters: the gait cycle duration, stance phase, swing phase, stride length and walking speed were computed for both sides to evaluate the timing of the patients’ ambulation;
- sEMG signals: the myoelectric activity of the eight muscles of interest of the operated limb was calculated to assess their activations during ambulation;
- sEMG features: the Root Mean Square (RMS) values were evaluated for each muscle of interest of the operated limb, whereas the Co-Contraction Index (CCI) values [29] were assessed for the agonist–antagonist muscle pairs RF/BF and TA/GL. Due to the different muscular activation timing during walking, RMS and CCI values were computed along the entire gait cycle, the stance phase, and the swing one;
- iEMG signals: the spontaneous and voluntary muscular activity of the functional flap was monitored in order to evaluate the eventual presence of fibrillation and motor units neurogenic recruitment;
- Clinical scales and questionnaires scores: the results of the clinical survey submitted to the patients were adopted for evaluating the functionality limitations during daily life, the post-operative neuropathic pain, and the QoL.

Except for the iEMG signals, all the indicators were computed for each patient in each evaluation session ($\textit{T}_{\textit{0}}$, $\textit{T}_{\textit{1}}$ and $\textit{T}_{\textit{2}}$) and compared with each other. Since the iEMG data were retrieved exclusively in the last evaluation session, they were used to deeply comprehend the activity of the functional flap and corroborate/falsify the hypothesis and discussions based on sEMG signals. Due to the reduced number of enrolled participants and their STS heterogeneity localization (i.e., thigh postero-medial, antero-lateral, and antero-medial compartments for patients P1, P2, and P3, respectively), a patient-specific analysis was carried out.

### Statistical analysis

The kinematic and sEMG signals are reported with respect to the gait cycle percentage with the vertical lines indicate the duration of the stance phase. The mean values and the standard deviations are represented as continuous lines and shaded areas, respectively. The other synthetic indicators were reported in boxplots, where the horizontal line denotes the median value, the lower and upper hinges correspond to the 25^th^ and 75^th^ percentiles, the whiskers extend from the hinge to the most extreme data points (i.e., no more than 1.5$\times$ interquartile range), and the + signs indicate the outliers.

The Shapiro-Wilk test was adopted for evaluating the normality of data distribution related to the computed ROMs, spatio-temporal parameters, sEMG features. Since it was non-Gaussian, the Wilcoxon signed-rank test was used to assess the eventual presence of statistically significant differences between such indicators evaluated in each session. Indeed, such a test can be used to compare two dependent and matched samples. Since three group data were encountered (i.e., $\textit{T}_{\textit{0}}$, $\textit{T}_{\textit{1}}$, and $\textit{T}_{\textit{2}}$) and three comparisons were carried out (i.e., $\textit{T}_{\textit{0}}$-$\textit{T}_{\textit{1}}$, $\textit{T}_{\textit{0}}$-$\textit{T}_{\textit{2}}$, $\textit{T}_{\textit{1}}$-$\textit{T}_{\textit{2}}$), Bonferroni correction was executed, and the significance level P was reduced from 0.05 to 0.0167.

## Results

### Lower limb kinematics

**Fig. 2:**
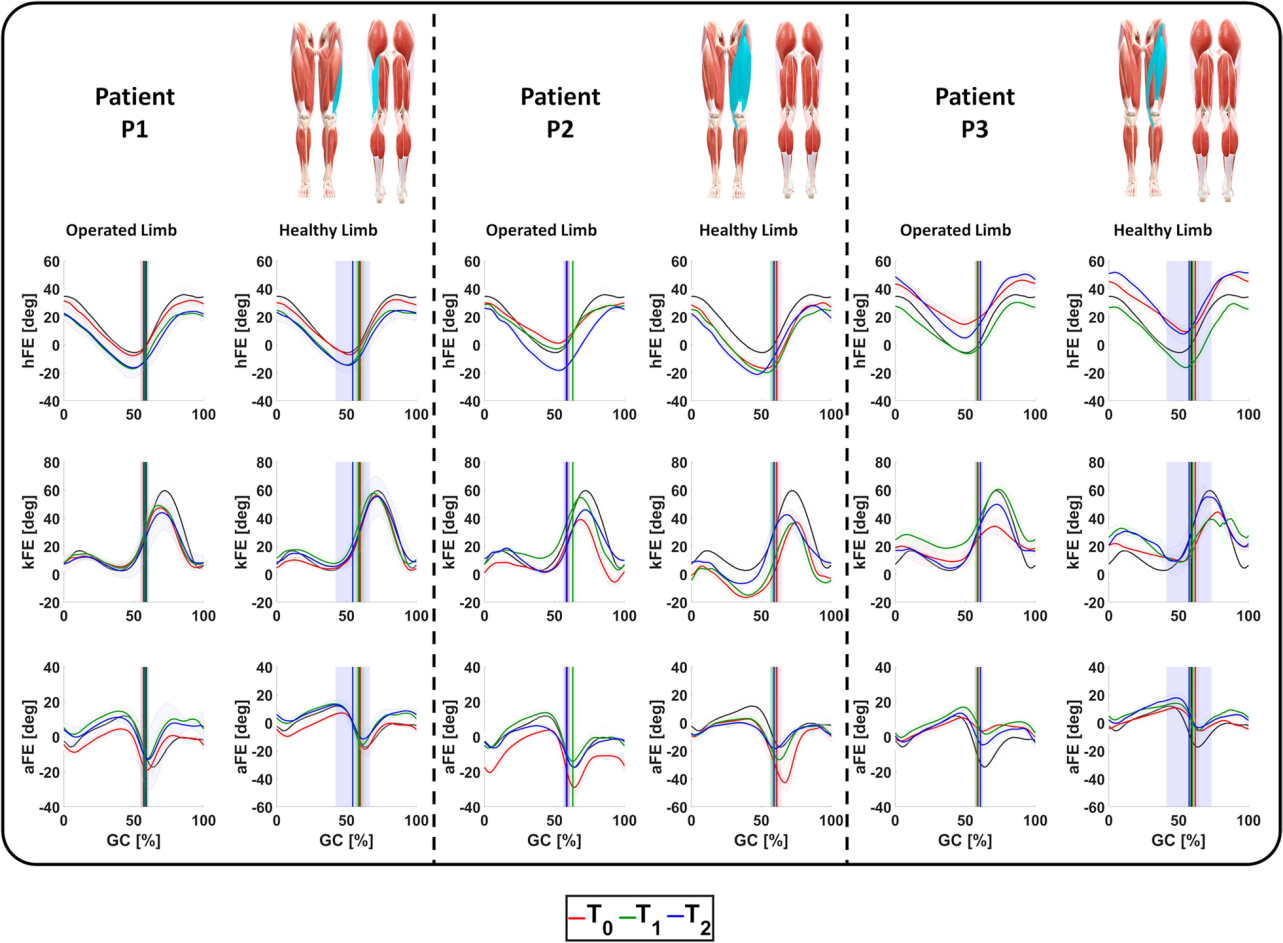
hFE, kFE, and aFE exerted during ambulation by the operated and healthy limb of the patients at $\textit{T}_{\textit{0}}$, $\textit{T}_{\textit{1}}$, and $\textit{T}_{\textit{2}}$ compared with age-related physiological values (black). For each patient, the anterior and posterior views of the lower limbs are reported with the muscles involved during surgery highlighted in light blue.

**Fig. 3:**
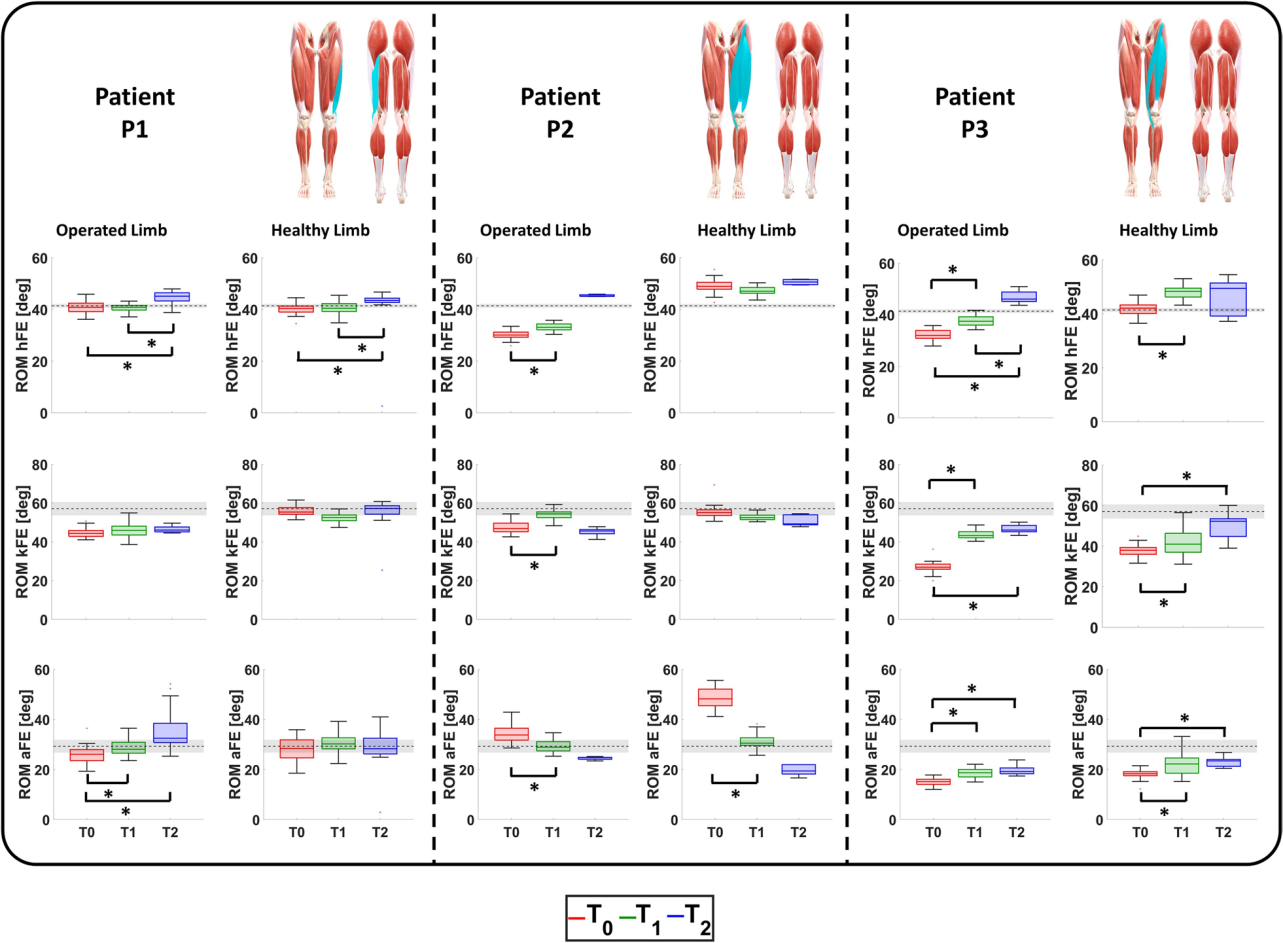
hROM, kROM, and aROM exerted during ambulation by the operated and healthy limb of the patients at $\textit{T}_{\textit{0}}$, $\textit{T}_{\textit{1}}$, and $\textit{T}_{\textit{2}}$ compared with age-related physiological values (black). For each patient, the anterior and posterior views of the lower limbs are reported with the muscles involved during surgery highlighted in light blue.

The hFE, kFE and aFE and the related ROMs of the patients’ healthy and operated limbs in the three evaluation sessions are represented in Figs. 2 and 3, respectively.

Patient P1: The rehabilitation treatment had no effect on kFE since only negligible differences were monitored among $\textit{T}_{\textit{0}}$, $\textit{T}_{\textit{1}}$, and $\textit{T}_{\textit{2}}$. Conversely, an increase in the hip extension at the end of the stance phase was encountered at $\textit{T}_{\textit{2}}$. This is evident from the ROM analysis, where statistically significant improvements were found between the sessions: from 40.81 deg at $\textit{T}_{\textit{0}}$ and $\textit{T}_{\textit{1}}$ to 45.12 deg at $\textit{T}_{\textit{2}}$ ($\hbox {P}_{{\textit{T}_{\textit{0}}\hbox {-} \textit{T}_{\textit{1}}}}<$0.001 and $\hbox {P}_{{\textit{T}_{\textit{0}}\hbox {-} \textit{T}_{\textit{2}}}}<$0.001).

The obtained results showed an improvement in aFE since an increase in the ankle flexion during the stance phase was encountered. This led to a statistically significant improvement in the ROM from 25.63 deg at $\textit{T}_{\textit{0}}$ to 28.12 deg at $\textit{T}_{\textit{1}}$ ($\hbox {P}_{{\textit{T}_{\textit{0}}\hbox {-} \textit{T}_{\textit{1}}}}=0.0015$) and 32.57 deg at $\textit{T}_{\textit{2}}$ ($\hbox {P}_{{\textit{T}_{\textit{0}}\hbox {-} \textit{T}_{\textit{2}}}}<$0.001).

As far as the healthy limb, the kFE and aFE demonstrated a physiological behavior along the three sessions. Conversely, the hFE and its ROM exhibited the same behavior of the operated one since statistically significant improvements were found between the sessions

Patient P2: The main effect of the proposed rehabilitation treatment was an alteration of hFE toward a more physiological behavior. This was due to an increase in hip extension in the late stance leading to a ROM increase: from 30.10 deg at $\textit{T}_{\textit{0}}$ to 33.10 deg at $\textit{T}_{\textit{1}}$ ($\hbox {P}_{{\textit{T}_{\textit{0}}\hbox {-} \textit{T}_{\textit{1}}}}<$0.001) and to 45.20 deg at $\textit{T}_{\textit{2}}$. As far as the knee, the rehabilitation led to an increase in the second peak of kFE during the swing phase and therefore a statistically significant improvement in its ROM (from 46.90 deg at $\textit{T}_{\textit{0}}$ to 54.40 deg at $\textit{T}_{\textit{1}}$, $\hbox {P}_{{\textit{T}_{\textit{0}}\hbox {-} \textit{T}_{\textit{1}}}}<$0.001). Nevertheless, this improvement was not sustained over time as the ROM observed at $\textit{T}_{\textit{2}}$ was comparable to that recorded at the beginning of the rehabilitation process.

As already observed in P1, the rehabilitation induced kinematic variations at the ankle too, suggesting a more global adaptation of lower limb movement. The obtained results showed that over time, the aFE tended toward a more physiological trend that was quantified in a reduction in aROM: from 33.80 deg at $\textit{T}_{\textit{0}}$ to 28.90 deg at $\textit{T}_{\textit{1}}$ $\hbox {P}_{{\textit{T}_{\textit{0}}\hbox {-} \textit{T}_{\textit{1}}}}<$0.001) and to 24.70 deg at $\textit{T}_{\textit{2}}$.

As for the healthy limb, similar variations were encountered in the aFE, while no modifications were encountered for the hFE and kFE.

Patient P3: The obtained results demonstrated progressive variations in hFE, kFE, and aFE toward more physiological patterns. Specifically, the hFE exhibited greater extension during the late stance phase just prior to toe-off; the kFE showed an increased second peak during the mid-swing phase and the aFE showed an improved plantarflexion during the transition from stance to swing phase. This was confirmed by the analysis of the related ROMs. The hROM moved from 32.00 deg at $\textit{T}_{\textit{0}}$ to 37.50 deg at $\textit{T}_{\textit{1}}$ ($\hbox {P}_{{\textit{T}_{\textit{0}}\hbox {-} \textit{T}_{\textit{1}}}}<$0.001) and 46.10 deg at $\textit{T}_{\textit{2}}$ ($\hbox {P}_{{\textit{T}_{\textit{0}}\hbox {-} \textit{T}_{\textit{2}}}}<$0.001, $\hbox {P}_{{\textit{T}_{\textit{1}}\hbox {-} \textit{T}_{\textit{2}}}}<$0.001). The kROM increased from 26.90 deg at $\textit{T}_{\textit{0}}$ to 43.20 deg at $\textit{T}_{\textit{1}}$ ($\hbox {P}_{{\textit{T}_{\textit{0}}\hbox {-} \textit{T}_{\textit{1}}}}<$0.001) and 46.10 deg at $\textit{T}_{\textit{2}}$ ($\hbox {P}_{{\textit{T}_{\textit{0}}\hbox {-} \textit{T}_{\textit{2}}}}<$0.001). The aROM improved from 15.10 deg at $\textit{T}_{\textit{0}}$ to 18.70 deg at $\textit{T}_{\textit{1}}$ ($\hbox {P}_{{\textit{T}_{\textit{0}}\hbox {-} \textit{T}_{\textit{1}}}}<$0.001) and 19.20 deg at $\textit{T}_{\textit{2}}$ ($\hbox {P}_{{\textit{T}_{\textit{0}}\hbox {-} \textit{T}_{\textit{2}}}}<$0.001).

The ambulation rehabilitation led to similar improvement in the healthy limb, too.

### Spatio-temporal parameters

**Fig. 4:**
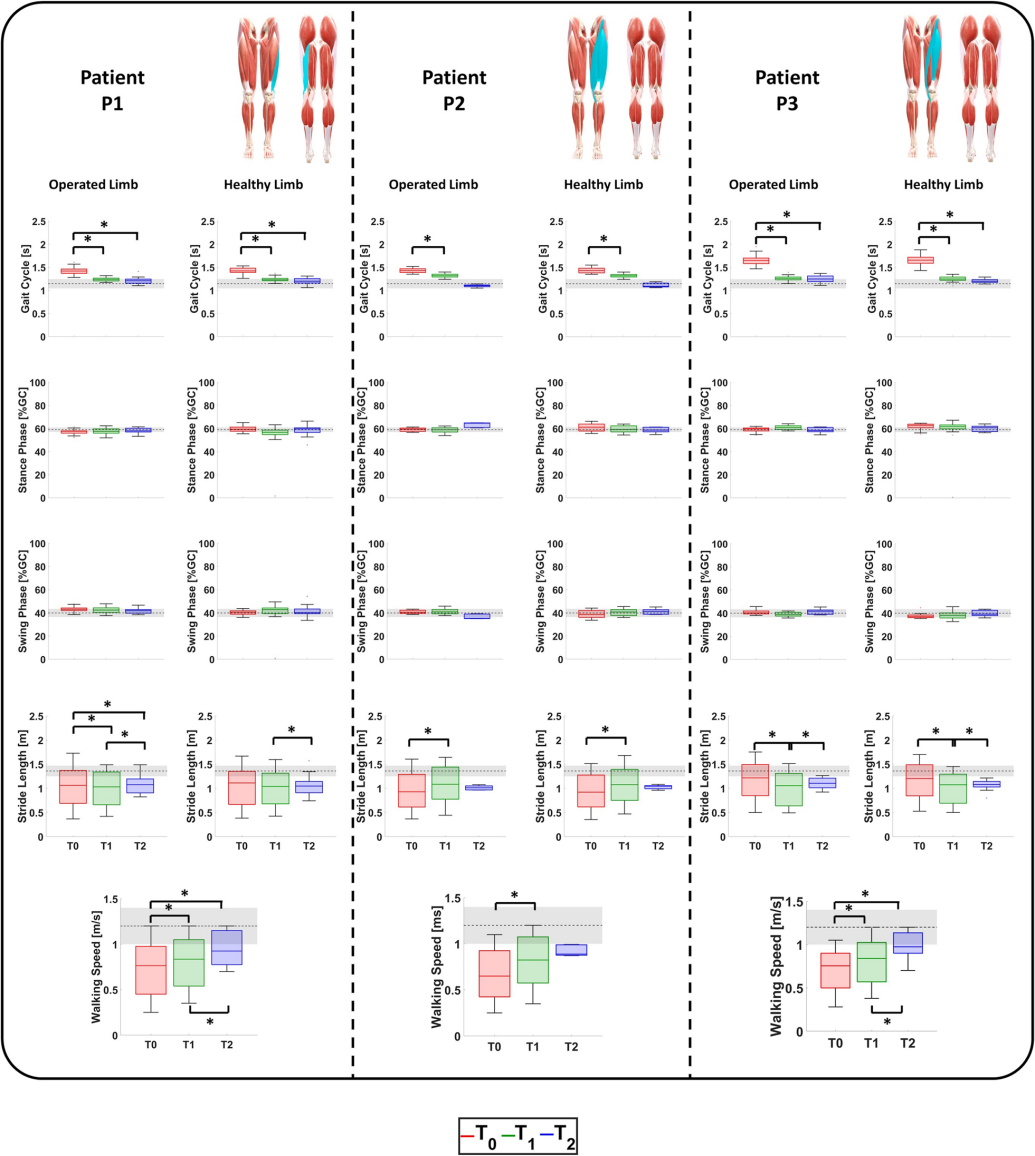
Spatio-temporal parameters of the operated and healthy limb of the patients at $\textit{T}_{\textit{0}}$, $\textit{T}_{\textit{1}}$, and $\textit{T}_{\textit{2}}$ compared with age-related physiological values (black). For each patient, the anterior and posterior views of the lower limbs are reported with the muscles involved during surgery highlighted in light blue.

The spatio-temporal parameters computed for both limbs in the three evaluation sessions are represented in Fig. 4.

Patient P1: The rehabilitation positively influenced the patient’s ambulation performance since walking speed increased from 0.7 m/s at $\textit{T}_{\textit{0}}$ to 0.8 m/s at $\textit{T}_{\textit{1}}$ ($\hbox {P}_{{\textit{T}_{\textit{0}}\hbox {-} \textit{T}_{\textit{1}}}}<$0.001) and to 0.9 m/s at $\textit{T}_{\textit{2}}$ ($\hbox {P}_{{\textit{T}_{\textit{0}}\hbox {-} \textit{T}_{\textit{2}}}}<$0.001).

The positive effects of this improvement can also be encountered in the gait cycle duration and stride length of the operated limb. The former significantly decrease from 1.4 s at $\textit{T}_{\textit{0}}$ to 1.2 s at $\textit{T}_{\textit{1}}$ ($\hbox {P}_{{\textit{T}_{\textit{0}}\hbox {-} \textit{T}_{\textit{1}}}}<$0.001) and to 1.1 s at $\textit{T}_{\textit{2}}$ ($\hbox {P}_{{\textit{T}_{\textit{0}}\hbox {-} \textit{T}_{\textit{2}}}}<$0.001). Similarly, the latter significantly moved from 1.0 m at $\textit{T}_{\textit{0}}$ and at $\textit{T}_{\textit{1}}$ to 1.1 s at $\textit{T}_{\textit{2}}$ ($\hbox {P}_{{\textit{T}_{\textit{0}}\hbox {-} \textit{T}_{\textit{1}}}}=0.0084$ and $\hbox {P}_{{\textit{T}_{\textit{0}}\hbox {-} \textit{T}_{\textit{2}}}}$= $\hbox {P}_{{\textit{T}_{\textit{1}}\hbox {-} \textit{T}_{\textit{2}}}}=0.0038$).

Patient P2: The main effect of the rehabilitation was a progressive increase in the walking speed: it significantly increased from 0.65 m/s at $\textit{T}_{\textit{0}}$ to 0.83 m/s at $\textit{T}_{\textit{1}}$ ($\hbox {P}_{{\textit{T}_{\textit{0}}\hbox {-} \textit{T}_{\textit{1}}}}<$0.001) and 0.89 m/s at $\textit{T}_{\textit{2}}$.

This improvement in terms of walking speed decreased the gait cycle duration and increased the stride length toward a more physiological value. The former significantly diminished from 1.43 s at $\textit{T}_{\textit{0}}$ to 1.32 at $\textit{T}_{\textit{1}}$ ($\hbox {P}_{{\textit{T}_{\textit{0}}\hbox {-} \textit{T}_{\textit{1}}}}<$0.001) and 1.10 s at $\textit{T}_{\textit{2}}$. Likewise, the stride length significantly increased from 0.93 m at $\textit{T}_{\textit{0}}$ to 1.08 m at $\textit{T}_{\textit{1}}$ ($\hbox {P}_{{\textit{T}_{\textit{0}}\hbox {-} \textit{T}_{\textit{1}}}}<$0.001) and 1.10 m at $\textit{T}_{\textit{2}}$.

Patient P3: The primary effect of the rehabilitation was a progressive increase in walking speed: it significantly improved from 0.75 m/s at $\textit{T}_{\textit{0}}$ to 0.84 m/s at $\textit{T}_{\textit{1}}$ ($\hbox {P}_{{\textit{T}_{\textit{0}}\hbox {-} \textit{T}_{\textit{1}}}}<$0.001), reaching 0.98 m/s at $\textit{T}_{\textit{2}}$ ($\hbox {P}_{{\textit{T}_{\textit{0}}\hbox {-} \textit{T}_{\textit{2}}}}<$0.001 and $\hbox {P}_{{\textit{T}_{\textit{1}}\hbox {-} \textit{T}_{\textit{2}}}}<$0.001).

This enhancement in walking speed was accompanied by a reduction in gait cycle duration toward more physiological values. The gait cycle time significantly decreased from 1.65 s at $\textit{T}_{\textit{0}}$ to 1.26 s at $\textit{T}_{\textit{1}}$ ($\hbox {P}_{{\textit{T}_{\textit{0}}\hbox {-} \textit{T}_{\textit{1}}}}<$0.001) and further to 1.25 s at $\textit{T}_{\textit{2}}$ ($\hbox {P}_{{\textit{T}_{\textit{0}}\hbox {-} \textit{T}_{\textit{2}}}}<$0.001).

### Surface myoelectric activity

**Fig. 5:**
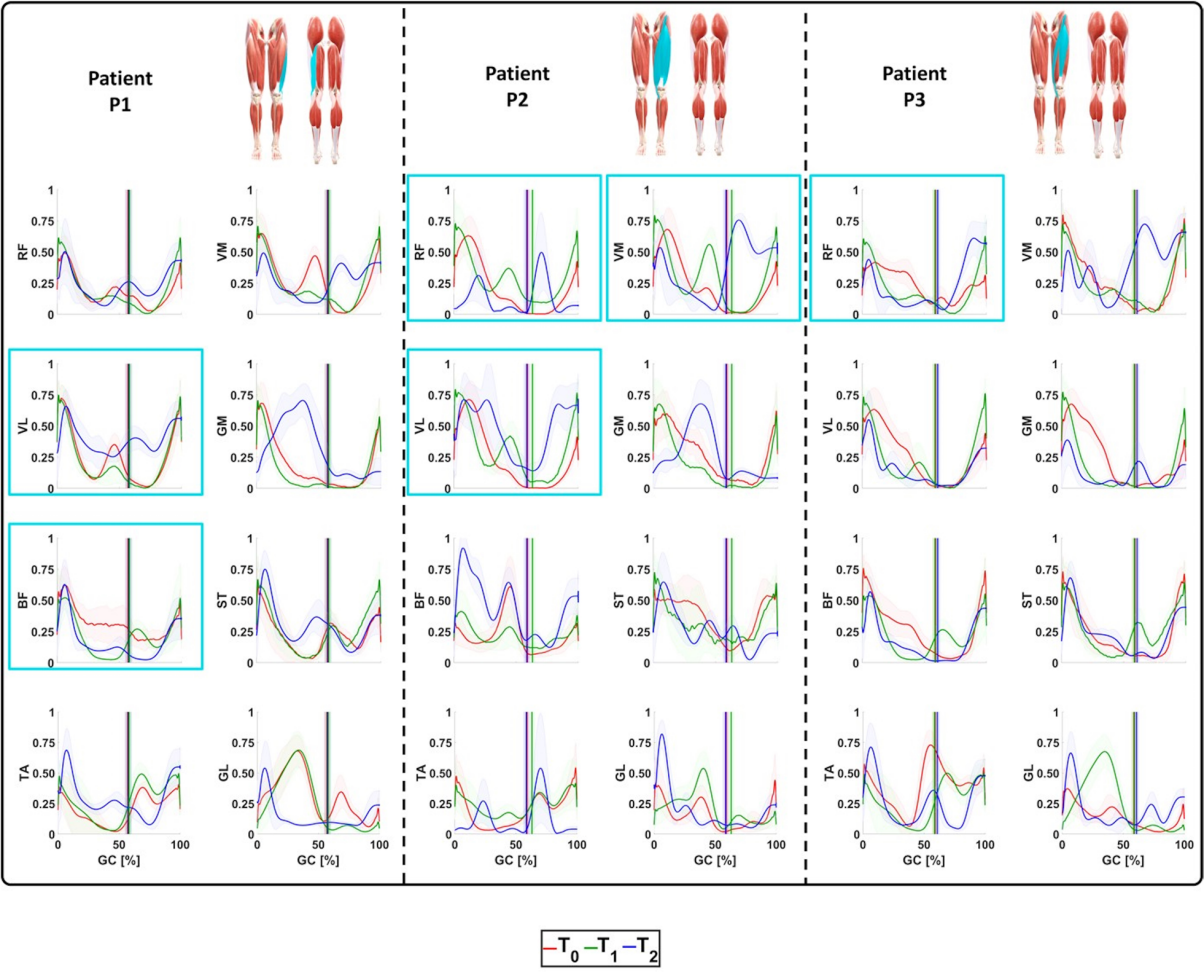
Activation of the selected muscles of the operated limb of the enrolled patients during ambulation at $\textit{T}_{\textit{0}}$, $\textit{T}_{\textit{1}}$, and $\textit{T}_{\textit{2}}$. For each patient, the anterior and posterior views of the lower limbs are reported with the muscles involved during surgery highlighted in light blue

**Fig. 6:**
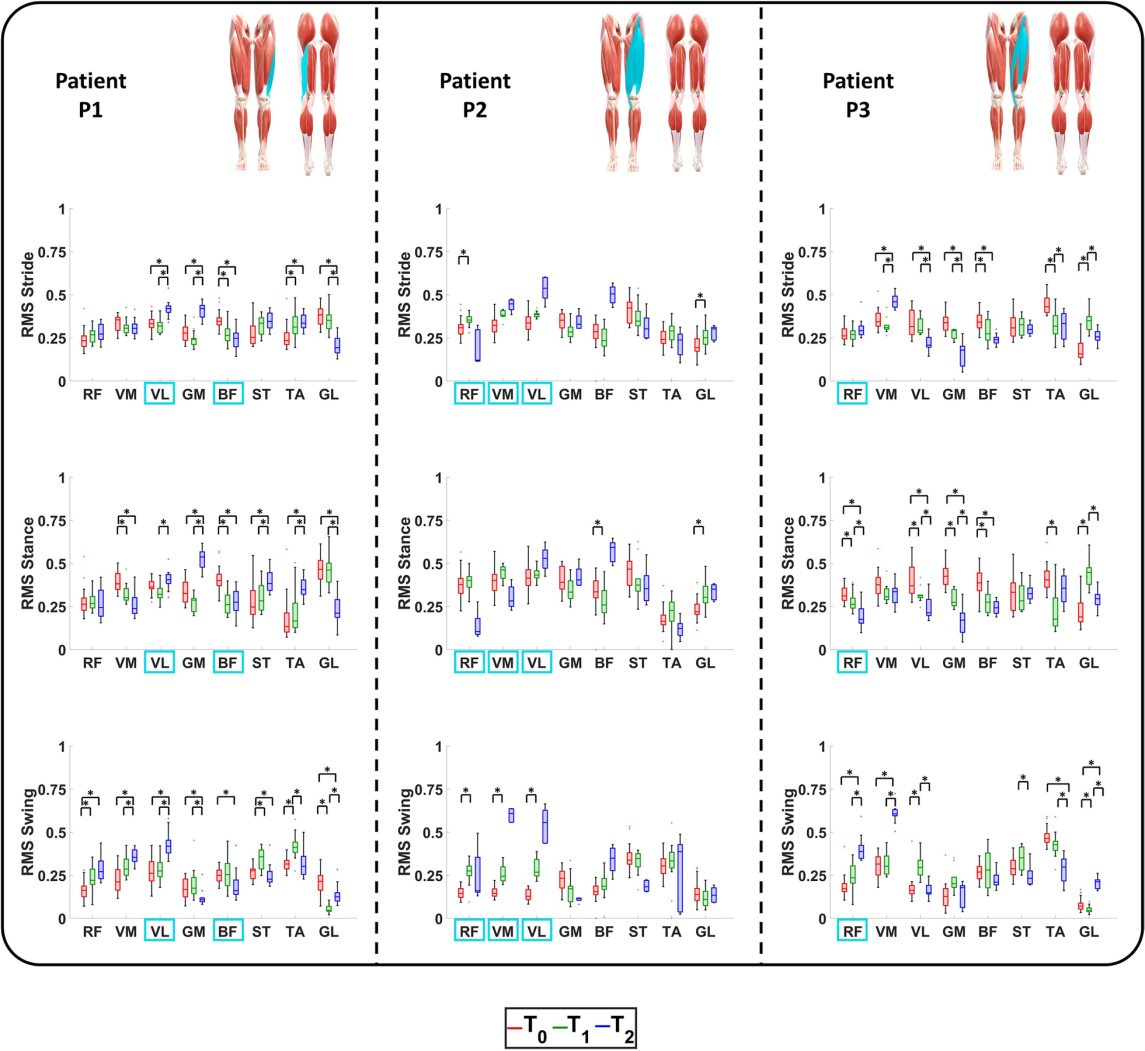
RMS values of the selected muscles of the operated limb of the enrolled patients during ambulation at $\textit{T}_{\textit{0}}$, $\textit{T}_{\textit{1}}$, and $\textit{T}_{\textit{2}}$. For each patient, the anterior and posterior views of the lower limbs are reported with the muscles involved during surgery highlighted in light blue

**Fig. 7:**
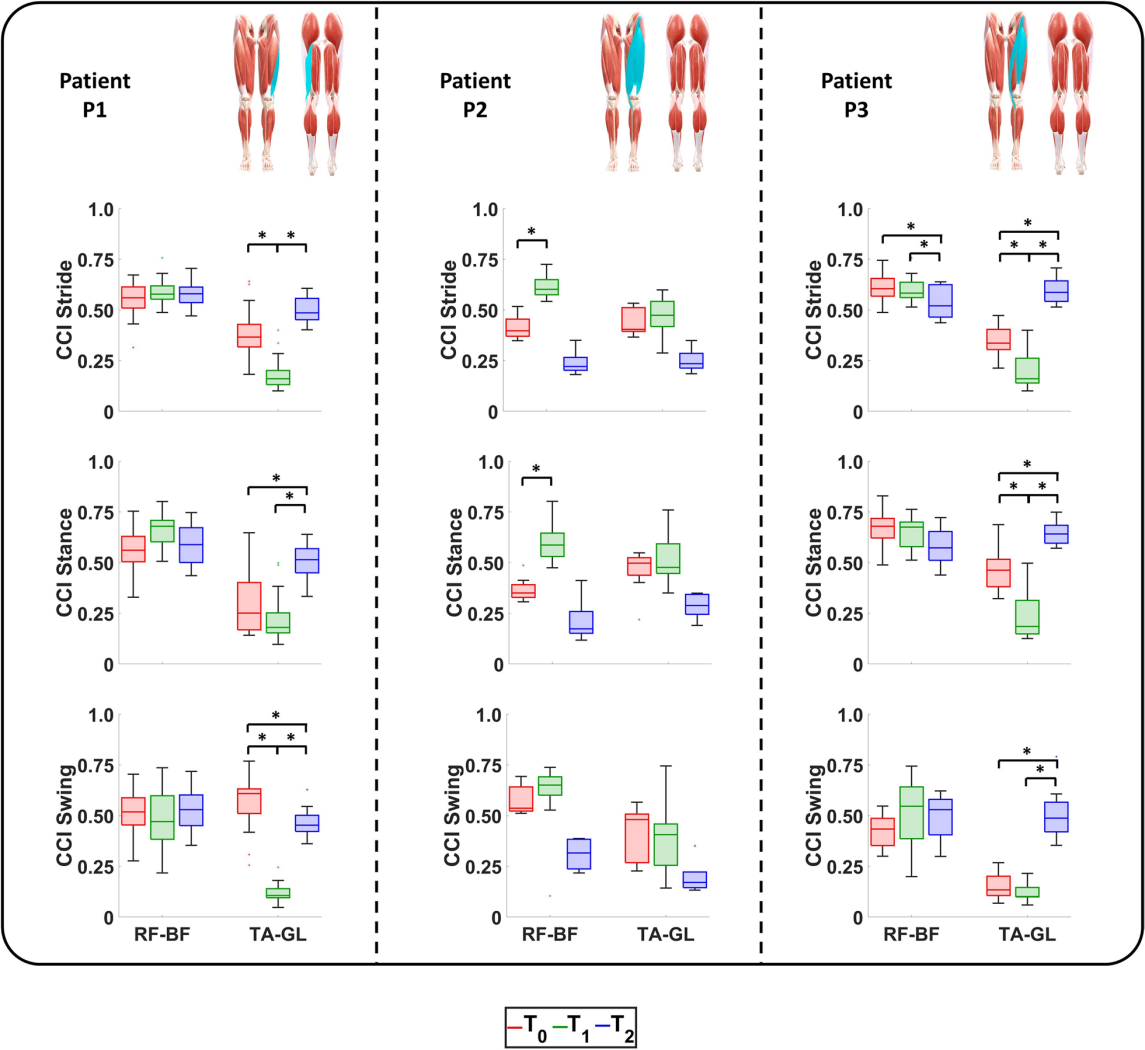
CCI values of the agonist–antagonist couple RF-BF and TA-GL of the operated limb of the enrolled patients during ambulation at $\textit{T}_{\textit{0}}$, $\textit{T}_{\textit{1}}$, and $\textit{T}_{\textit{2}}$. For each patient, the anterior and posterior views of the lower limbs are reported with the muscles involved during surgery highlighted in light blue

The sEMG signals of the muscles of interest are reported in Fig. 5, whereas the RMS and CCI values are represented in Figs. 6 and 7, respectively.

Patient P1: The obtained results demonstrated a physiological VL activation at $\textit{T}_{\textit{0}}$ and $\textit{T}_{\textit{1}}$. During the last evaluation session, a hyperactivation was found in terms of RMS values that moved from 0.33 at $\textit{T}_{\textit{0}}$ and 0.31 at $\textit{T}_{\textit{1}}$ to 0.42 at $\textit{T}_{\textit{2}}$. Nonetheless, this VL hyper-activation was counterbalanced by the adjacent muscles such as the VM and RF. Indeed, the VM showed a statistically significant reduction from the beginning of the rehabilitation to the follow-up in terms of RMS values. This is evident in the whole gait cycle (from 0.36 at $\textit{T}_{\textit{0}}$ to 0.31 at $\textit{T}_{\textit{1}}$ and to 0.30 at $\textit{T}_{\textit{2}}$) and statistically significant during the stance phase (from 0.38 at $\textit{T}_{\textit{0}}$ to 0.30 at $\textit{T}_{\textit{1}}$, $\hbox {P}_{{\textit{T}_{\textit{0}}\hbox {-} \textit{T}_{\textit{1}}}}=0.0125$, and to 0.24 at $\textit{T}_{\textit{2}}$ $\hbox {P}_{{\textit{T}_{\textit{0}}\hbox {-} \textit{T}_{\textit{2}}}}<$0.0125). Conversely, the RF maintained a physiological activation throughout the whole study [30], showing negligible differences among the sessions. As far as the BF, its activation during the last evaluation session was almost physiological since it was mainly active during the early stance and terminal swing [30]. This was due to a significant reduction in RMS values in the whole gait cycle: from 0.35 at $\textit{T}_{\textit{0}}$ to 0.26 at $\textit{T}_{\textit{1}}$ and to 0.25 at $\textit{T}_{\textit{2}}$ ($\hbox {P}_{{\textit{T}_{\textit{0}}\hbox {-} \textit{T}_{\textit{1}}}}$=$\hbox {P}_{{\textit{T}_{\textit{0}}\hbox {-} \textit{T}_{\textit{2}}}}<$0.001). The GM was characterized by a physiological behavior at the beginning and at the end of the rehabilitation [30]. Nevertheless, during the long-term follow-up session, it exhibited an alteration because of its less activation during the whole stance phase. This was evident from the analysis of the RMS values: from 0.33 at $\textit{T}_{\textit{0}}$ and from 0.28 at $\textit{T}_{\textit{1}}$ to 0.54 at $\textit{T}_{\textit{2}}$ ($\hbox {P}_{{\textit{T}_{\textit{0}}\hbox {-} \textit{T}_{\textit{1}}}}$=$\hbox {P}_{{\textit{T}_{\textit{0}}\hbox {-} \textit{T}_{\textit{2}}}}<$0.001). No essential variations were found for the ST activation.

As previously mentioned, although the surgery primarily affected the biomechanics of the hip and knee, it also had an impact on the ankle. The obtained results demonstrated a physiological behavior for the TA activation among the whole sessions [30], while an alteration of GL one was monitored. Nevertheless, a significant reduction in CCI values of the couple TA/GL during swing phase was retrieved: from 0.61 at $\textit{T}_{\textit{0}}$ to 0.45 at $\textit{T}_{\textit{2}}$ ($\hbox {P}_{{\textit{T}_{\textit{0}}\hbox {-} \textit{T}_{\textit{2}}}}=0.0097$).

Patient P2: The obtained results demonstrated how the RF activation recorded in the last evaluation session can be compared with the physiological one in terms of intensity and timing [30]. This was caused by a reduction in activation in terms of RMS values computed for the stride: from 0.31 at $\textit{T}_{\textit{0}}$ to 0.35 at $\textit{T}_{\textit{1}}$ ($\hbox {P}_{{\textit{T}_{\textit{0}}\hbox {-} \textit{T}_{\textit{1}}}}=0.0063$) and 0.12 at $\textit{T}_{\textit{2}}$. As far as the swing phase, the obtained results demonstrated VL and VM activation patterns were comparable with the physiological ones [30]. This was reached thanks to an increase in muscular activation over time and can be observed in the RMS values of the swing phase. In case of VL, they moved from 0.13 at $\textit{T}_{\textit{0}}$ to 0.27 at $\textit{T}_{\textit{1}}$ ($\hbox {P}_{{\textit{T}_{\textit{0}}\hbox {-} \textit{T}_{\textit{1}}}}=0.0020$) and 0.56 at $\textit{T}_{\textit{2}}$. In case of VM, they moved from 0.15 at $\textit{T}_{\textit{0}}$ to 0.24 at $\textit{T}_{\textit{1}}$ ($\hbox {P}_{{\textit{T}_{\textit{0}}\hbox {-} \textit{T}_{\textit{1}}}}=0.0020$) and 0.61 at $\textit{T}_{\textit{2}}$. As far as the stance phase, although the VM activation was not relevant, it was counter-balanced by that of VL.

For what concerns the hamstrings muscles, at the long-term follow-up sessions, they presented physiological trends with the GM and BF cases being noteworthy. The GM remained active throughout the entire stance phase, rather than being limited to its typical activation at initial contact [30]. In addition, the monitored BF activation over-time demonstrated the efficacy of the rehabilitation protocol since a gradual increase in its amplitude in the stance phase was encountered: from 0.33 at $\textit{T}_{\textit{0}}$ to 0.26 at $\textit{T}_{\textit{1}}$ ($\hbox {P}_{{\textit{T}_{\textit{0}}\hbox {-} \textit{T}_{\textit{1}}}}=0.0027$) and 0.59 at $\textit{T}_{\textit{2}}$. This recovery, combined with that of RF, led to a significant reduction in their CCI values along the whole gait cycle: from 0.40 at $\textit{T}_{\textit{0}}$ to 0.60 at $\textit{T}_{\textit{1}}$ ($\hbox {P}_{{\textit{T}_{\textit{0}}\hbox {-} \textit{T}_{\textit{1}}}}=0.0039$) and 0.22 at $\textit{T}_{\textit{2}}$.

Regarding the ankle’s muscles, at $\textit{T}_{\textit{2}}$ the TA presented an activation pattern comparable with the physiological one in terms of amplitude but delayed in terms of time. [30] Conversely, the GL presented the same alterations already encountered in the patient P1. Nonetheless, the rehabilitation treatment led to a decrease in their CCI values computed along the gait cycle from 0.40 at $\textit{T}_{\textit{0}}$ to 0.47 at $\textit{T}_{\textit{1}}$ and 0.23 at $\textit{T}_{\textit{2}}$.

Patient P3: The obtained results demonstrated how, during the last evaluation session, the RF showed a physiological behavior [30]. This result was possible thanks to a reduction in the sEMG signal during the stance phase from the beginning to the long-term follow-up. This was quantified in terms of RMS values that moved from 0.31 at $\textit{T}_{\textit{0}}$ to 0.26 at $\textit{T}_{\textit{1}}$ ($\hbox {P}_{{\textit{T}_{\textit{0}}\hbox {-} \textit{T}_{\textit{1}}}}=0.0080$) and 0.17 at $\textit{T}_{\textit{2}}$($\hbox {P}_{{\textit{T}_{\textit{0}}\hbox {-} \textit{T}_{\textit{2}}}}<$0.001 and $\hbox {P}_{{\textit{T}_{\textit{1}}\hbox {-} \textit{T}_{\textit{2}}}}=0.0137$). In the follow-up the VM demonstrated a hyperactivation with respect to the beginning and the end of the rehabilitation: from 0.32 at $\textit{T}_{\textit{0}}$ to 0.30 at $\textit{T}_{\textit{1}}$ and 0.61 at $\textit{T}_{\textit{2}}$ ($\hbox {P}_{{\textit{T}_{\textit{0}}\hbox {-} \textit{T}_{\textit{2}}}}<$0.001 and $\hbox {P}_{{\textit{T}_{\textit{1}}\hbox {-} \textit{T}_{\textit{2}}}}=0.0020$). This phenomenon was able to counterbalance the ipo-activation of the adjacent VL.

As far as the hamstrings muscles, at the long-term follow-up sessions they presented physiological trends with the BF case being noteworthy. Indeed, the combined physiological activation of BF and RF at $\textit{T}_{\textit{2}}$ led to a significant reduction in the CCI values along the whole gait cycle: from 0.61 at $\textit{T}_{\textit{0}}$ to 0.58 at $\textit{T}_{\textit{1}}$ and 0.52 at $\textit{T}_{\textit{2}}$ ($\hbox {P}_{{\textit{T}_{\textit{0}}\hbox {-} \textit{T}_{\textit{2}}}}=0.0049$ and $\hbox {P}_{{\textit{T}_{\textit{1}}\hbox {-} \textit{T}_{\textit{2}}}}=0.0049$).

Concerning the sEMG ankle’s muscles signals monitored at the last evaluation session, although the GL still presented some alterations, the TA regained its typical behavior.

### Invasive myoelectric activity

**Fig. 8:**
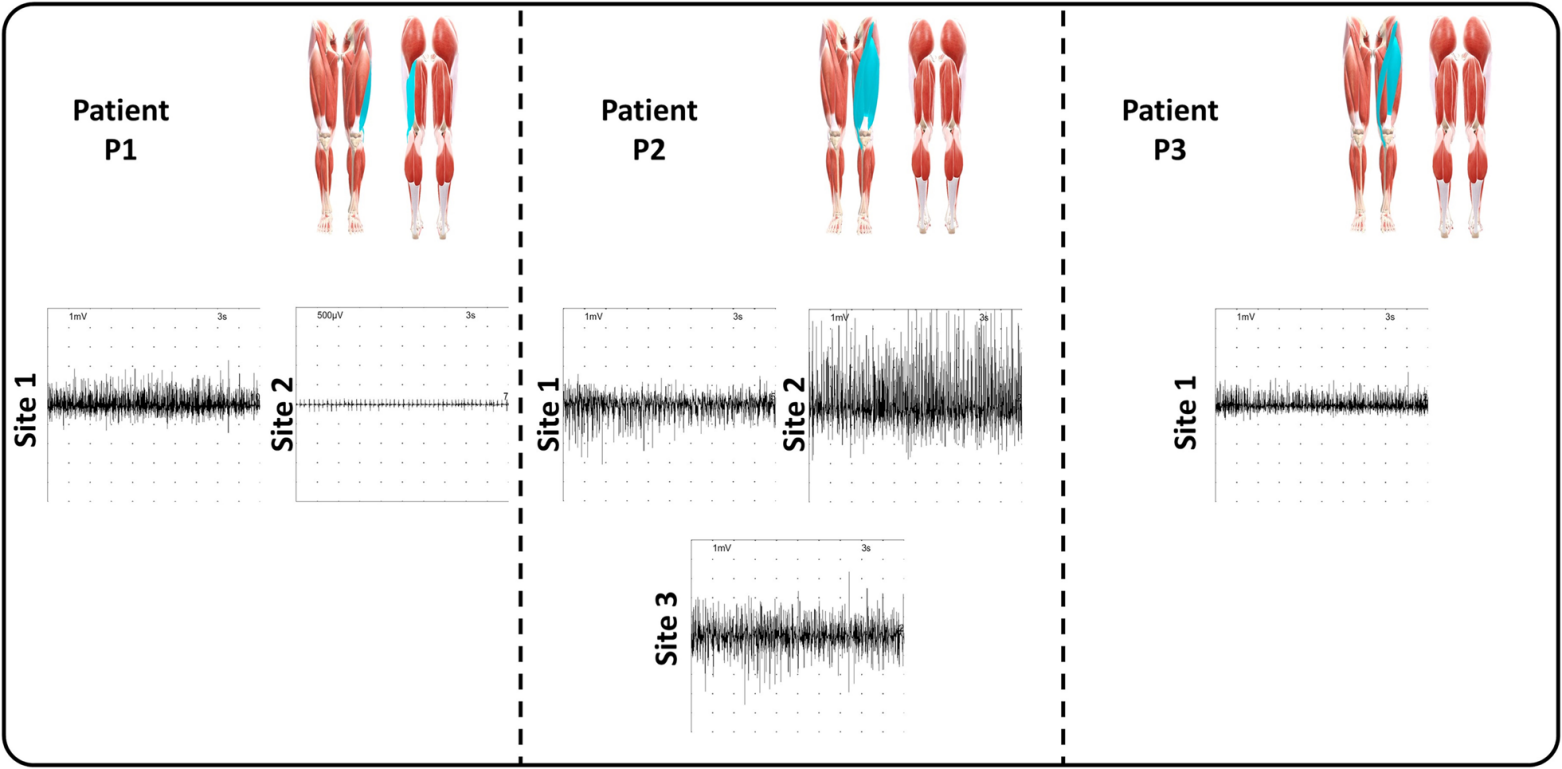
Voluntary activation of the functional flap of the enrolled patients evaluated via needle electromyography at $\textit{T}_{\textit{2}}$. For each patient, the anterior and posterior views of the lower limbs are reported with the muscles involved during surgery highlighted in light blue

The iEMG signals recorded in the last evaluation session are reported in Fig. 8.

Patient P1: In most of the explored sites, the flap exhibited a physiological motor unit recruitment with no evidence of fibrillation potentials, suggesting a complete reinnervation process. Nonetheless, fibrillation potentials were detected at specific points accompanied by a neurogenic recruitment pattern with single motor units with a low amplitude indicative of axonal damage and an ongoing reinnervation process.

Patient P2: The flap revealed a physiological pattern of motor unit recruitment, with no signs of spontaneous activity, such as fibrillation potentials. These results indicated an adequate reinnervation and therefore the quadriceps compartment achieved a good degree of neuromuscular recovery.

Patient P3: The iEMG signals demonstrated occasional fibrillation potentials, suggesting mild ongoing denervation activity. Nevertheless, the muscle exhibited a good motor unit recruitment pattern, indicating a relatively recovered functional capacity.

### Clinical survey

**Fig. 9:**
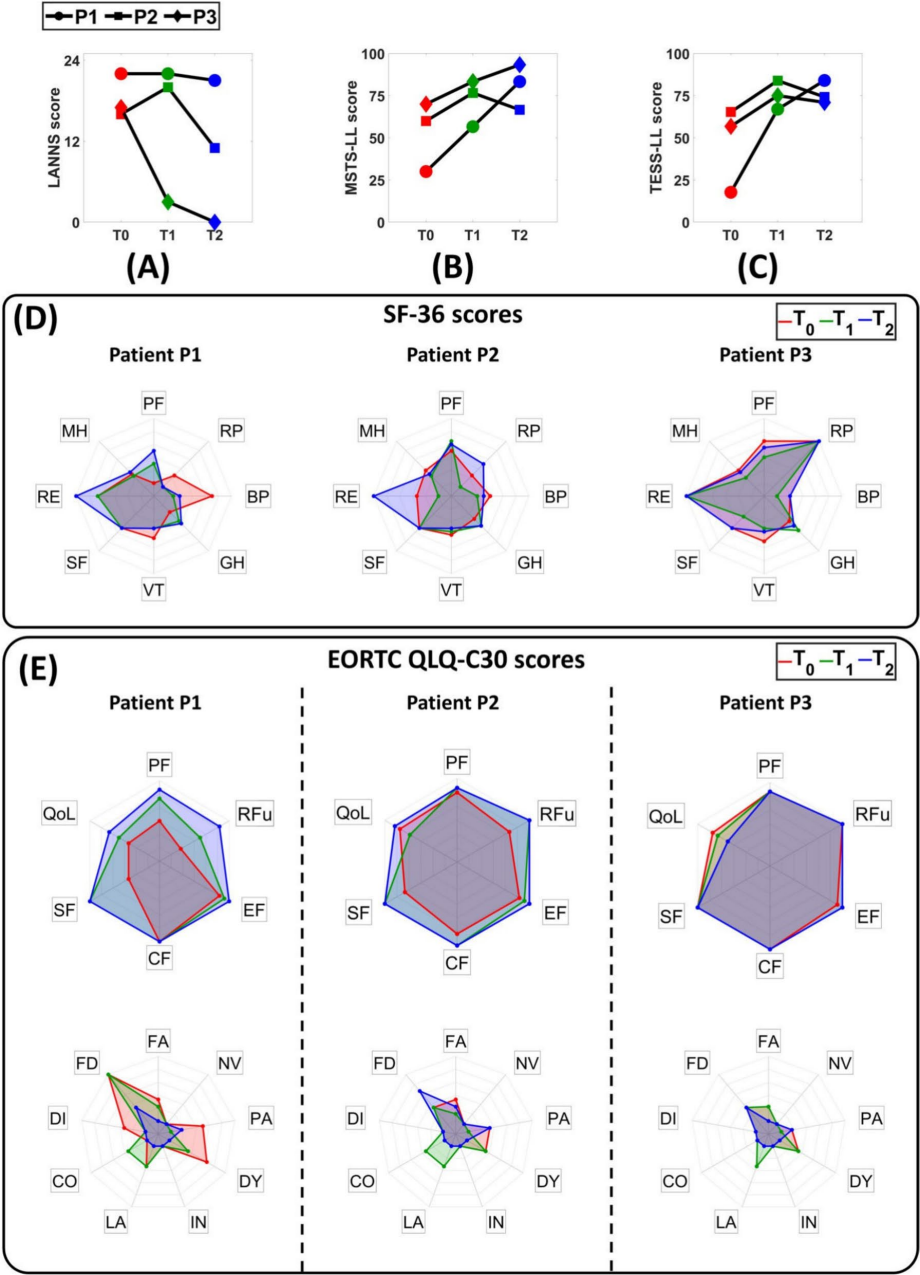
LANNS (**A**), MSTS-LL (**B**), TESS-LL (**C**), SF-36 (**D**), and EORTC QLQ-C30 (**E**) scores obtained by the enrolled patients at $\textit{T}_{\textit{0}}$, $\textit{T}_{\textit{1}}$, and $\textit{T}_{\textit{2}}$.* BP* Bodily Pain,* CF* Cognitive Function,* CO* Constipation,* DI* Diarrhea,* DY* Dyspnea,* GH* General Health,* EF* Emotional Function,* FA* Fatigue,* FD* Financial Difficulties,* IN* Insomnia,* LA* Lost of Appetite,* MH* Mental Health,* NV* Nausea and Vomiting,* PA* Pain,* PF* Physical Function,* RE* Role Emotional,* RFu* Role Function,* RP* Role Physical,* SF* Social Functioning,* VT* Vitality

The results of the clinical survey are reported in Fig. 9.

Patient P1: Although the intensity of post-operative neuropathic pain remained essentially stable, as indicated by the LANSS scores (22 at $\textit{T}_{\textit{0}}$ and $\textit{T}_{\textit{1}}$, 21 at $\textit{T}_{\textit{2}}$), notable improvements were observed in physical functionality and activity-related limitations. Specifically, the MSTS-LL score increased from 30% at $\textit{T}_{\textit{0}}$ to 52% at $\textit{T}_{\textit{1}}$ and 83% at $\textit{T}_{\textit{2}}$, while the TESS-LL score improved from 16% at $\textit{T}_{\textit{0}}$ to 67% at $\textit{T}_{\textit{1}}$ and 84% at $\textit{T}_{\textit{2}}$. These results suggested the restoration of excellent functionalities despite the persistence of neuropathic pain.

As far as the SF-36 scores, they demonstrated an overall improvement in the perceived QoL perceived by the patient. This was evident in several domains of the scale especially in the physical functioning, role emotional, mental and general health. A similar trend was also observed in the EORTC-QLQ-C30 scores. Indeed, all the symptoms disappeared, except for the pain and the financial difficulties which reduced considerably: from 50 at $\textit{T}_{\textit{0}}$ to 0 at $\textit{T}_{\textit{1}}$ and 17 at $\textit{T}_{\textit{2}}$ for the former, whereas from 100 at $\textit{T}_{\textit{0}}$ and $\textit{T}_{\textit{1}}$ to 33 at $\textit{T}_{\textit{2}}$ for the latter.

Patient P2: The obtained results demonstrated an essential reduction in post-operative neuropathic pain quantified in terms of LANSS score: from 16 at $\textit{T}_{\textit{0}}$ to 11 at $\textit{T}_{\textit{2}}$. Meanwhile, an improvement in physical functionality was observed: MSTS-LL and TESS-LL scores moved from 60% at $\textit{T}_{\textit{0}}$ to 77% at $\textit{T}_{\textit{1}}$ and from 65% at $\textit{T}_{\textit{0}}$ to 84% at $\textit{T}_{\textit{1}}$, respectively. Notably, these gains were maintained at the long-term follow-up indicating sustained functional recovery.

Concerning the perceived QoL, the SF-36 and EORTC-QLQ-C30 scores were in line, demonstrating a substantial improvement in almost all their domains. In addition, at $\textit{T}_{\textit{2}}$ all the symptoms disappeared, except for the fatigue, pain, and financial difficulties.

Patient P3: The rehabilitation led to a drastic reduction in neuropathic pain quantified in terms of LANSS scores that moved from 17 at $\textit{T}_{\textit{0}}$ to 3 at $\textit{T}_{\textit{1}}$ and 0 at $\textit{T}_{\textit{2}}$. This was correlated to an increase in physical functionality from good to excellent in terms of MSTS-LL scores (70% at $\textit{T}_{\textit{0}}$ and 93% at $\textit{T}_{\textit{2}}$) and from moderate to good in terms of TESS-LL scores (57% at $\textit{T}_{\textit{0}}$ and 71% at $\textit{T}_{\textit{2}}$).

The SF-36 and EORTC-QLQ-C30 scores demonstrated an improvement over-time of their domains except for the physical functioning, vitality, and QoL. Nonetheless, at $\textit{T}_{\textit{2}}$ all the symptoms disappeared, except for the pain and financial difficulties.

## Discussion

Patient P1: The patient underwent a STS surgical resection involving the BF and VL, which were entirely removed and replaced with a functional flap from the latissimus dorsi. As far as the BF, the iEMG and sEMG signals demonstrated a physiological motor unit recruitment and myoelectric activity with a mild fibrillation potential. Concerning the VL, the iEMG signals revealed no signs of denervation and fibrillation potential: this suggests that the FFMT was effective in the restoration of functional capability. The sEMG signals were characterized by a marked hyperactivation, especially during the long-term follow-up. Nonetheless, this appeared to compensate for the reduced contribution of the VM. The onset of compensatory strategies were further supported by the GM activation pattern, which remained active throughout the entire stance phase, rather than being limited to its typical early activation. This adaptation likely played a stabilizing role in response to proximal muscle deficits and demonstrates the body’s ability to reorganize motor control to preserve gait stability.

These neuromuscular adaptations were reflected in the operated limb’s hFE improvements and also in the aFE, which was not directly targeted by the surgery or rehabilitation. Notably, improvements were also encountered in the healthy limb’s hFE indicating a broader systemic effect of the rehabilitative protocol. Moreover, the patient exhibited enhanced walking speed, increased stride length and reduced gait cycle duration. These findings highlighted the efficacy of the rehabilitation program, not only in addressing localized deficits but also in promoting global gait reorganization.

From a clinical perspective, although neuropathic pain remained stable over time, the improvement in terms of kinematic and myoelectric activity led to substantial improvements in functional ability and perceived quality of life.

Patient P2: The patient underwent surgical resection involving partial removal of the RF, VL and VM. The needle electromyography at the long-term follow-up revealed no evidence of fibrillation potentials, in any of the examined muscles indicating effective reinnervation and a satisfactory recovery of muscle function. These results were corroborated by the sEMG signals retrieved during gait analyses. The RF showed a normal activation profile, while the VM activation was relatively limited during the stance phase. Its contribution appeared to be functionally compensated by an increased VL activation, maintaining appropriate quadriceps function during the loading response and early stance. The BF progressively recovered over time and, as seen in patient P1, the GM was active throughout the stance phase, deviating from its typical temporal pattern. This sustained activation likely reflected a compensatory strategy adopted by the patient to enhance pelvic and lower limb stability during ambulation. Although some alterations were noted in TA and GL, their coordination allowed for a smooth gait without excessive co-contraction.

These neuromuscular adaptations were mirrored by progressive improvements in joint kinematics, especially in the operated limb’s hFE, kFE, and aFE. Interestingly, a significant functional enhancement was also observed in the healthy limb’s ankle, suggesting that the rehabilitation program exerted bilateral benefits on gait control and neuromuscular efficacy. This finding reinforced the hypothesis that a well-designed, intensive rehabilitation protocol can promote global gait reorganization, even in the presence of localized surgical interventions.

The clinical analysis demonstrated substantial improvements in functional status, as evidenced by the MSTS-LL and TESS-LL scores, a reduction in neuropathic pain, and a considerable improvement in the QoL perception.

Patient P3: The patient underwent extensive surgical resection involving the complete removal of the RF with functional reconstruction using a functional flap from the contralateral thigh. The iEMG signals at the long-term follow-up revealed occasional fibrillation potentials, indicating residual signs of denervation. Nevertheless, this was not functionally limiting, as the sEMG signals demonstrated a physiological activation pattern. The VM and VL exhibited complementary activation, effectively compensating for each other’s variations in activity. Notably, the VM demonstrated increased activation over time, particularly during the stance phase, possibly to counterbalance the reduced contribution of the VL, thereby preserving global quadriceps function. The hamstrings muscles presented physiological activation patterns, suggesting no major neuromotor disruptions occurred in that compartment.

At the kinematic level, the patient demonstrated progressive and consistent improvements across all lower limb joints of both limbs. These changes reflect a more efficient and coordinated gait pattern and are indicative of successful functional adaptation and recovery following both surgery and rehabilitation.

Clinically, a noteworthy outcome was the complete resolution of neuropathic pain, although some functional limitations and minor symptoms remained.

The aforementioned patient-specific findings highlighted both the heterogeneity and the consistency of neuromuscular recovery following STS resection and FFMT. The three cases differed in terms of STS location and extent and stressed the need for recovery timelines tailored according to the anatomical and functional consequences of the surgery rather than being defined on a uniform rehabilitation protocol.

Nonetheless, several broader conclusions can be drawn.

The iEMG signals and the presence of coherent, task-specific sEMG activity during all the patients’ gaits at long-term follow-up confirmed literature findings on the need of 12 months for reaching a complete or quasi-complete reinnervation of the reconstructed or preserved muscles [31]. In line with existing literature on FFMT [11, 13], functional reinnervation unfolded over a prolonged timescale and was accompanied by progressive refinement of motor unit recruitment and inter-muscular coordination. Noteworthy, the proposed study was the first one deeply analyzing in a quantitative manner the effects of FFMT on muscular activation. The obtained results demonstrated how recovery did not consist of a return to preoperative activation timing but rather involved the onset of adaptive motor patterns that effectively supported joint kinematics and ambulation performance.

Within this framework, coordinated interactions between adjacent muscles emerged as a mechanism supporting joint function, particularly at the knee. All the patients demonstrated functional synergy VL and VM, whereby complementary activation patterns compensated for local deficits and contributed to stable and efficient knee extension during stance. Rather than reflecting isolated muscle recovery, this behavior suggests a reorganization of quadriceps motor control toward synergistic solutions that preserve joint mechanics despite partial muscle loss or reconstruction. Such inter-muscular coordination was likely reinforced through task-specific rehabilitation and represented a key determinant of functional knee stability following FFMT.

Similarly, the GM prolonged activation observed in patients P1 and P2 should be interpreted as a functional compensatory strategy rather than a pathological alteration. Following extensive muscle loss or reconstruction, sustained proximal muscle activation likely enhanced hip and pelvic stability during stance, facilitating load acceptance and limb control. Comparable proximal stabilization strategies have been reported in gait adaptations associated with muscle weakness or altered biomechanical demands, supporting the interpretation of this pattern as an effective, though non-normative, motor solution.

Moreover, a relevant finding was the presence of kinematic and neuromuscular improvements in the healthy limb, observed in patients P1 and P3. This bilateral adaptation suggests that the rehabilitation protocol induced a system-level motor function reorganization rather than isolated recovery of a muscle segment of the operated limb. The intensity and task-specific structure of the rehabilitation program likely promoted symmetrical gait strategies and improved inter-limb coordination.

Since all the previous results were corroborated by the retrieved clinical scales and patient-oriented questionnaires scores, it is important to emphasize that, despite the three reported cases being markedly different from one another, all patients exhibited a reduction in pain, an improvement in motor function, and an enhancement of quality of life.

## Conclusions

The aim of the study was to evaluate the effect of a customized rehabilitation protocol on walking capabilities of three patients with LL-STS treated with radical resection and FFMT. Instrumental measures (i.e., an optoelectronic system, surface electromyography sEMG and an invasive one iEMG) were adopted to evaluate the patients’ ambulation performance at the beginning ($\textit{T}_{\textit{0}}$), at the end ($\textit{T}_{\textit{1}}$) of the rehabilitation, and at a long-term follow-up ($\textit{T}_{\textit{2}}$). In addition, clinical scales and patient-oriented questionnaires were used to assess the abilities in daily life, the post-operative neuropathic pain, and the perceived QoL.

Although the proposed study was limited by the small sample size of the enrolled patients and their STS heterogeneity localization (i.e., thigh postero-medial, antero-lateral and antero-medial compartment), all the patients exhibited notable neuromuscular recovery, as reflected by improvements in both muscle activation patterns and lower limb joint kinematics. A consistent observation across patients was the presence of compensatory activation patterns that enhanced lower limb stability. In several cases, synergistic interactions between anatomically or functionally related muscles contributed to the preservation of joint function (e.g., hyperactivation of the GM or coordinated activity between the VL and VM). The rehabilitation program also promoted progressive improvements in joint motion and spatio-temporal parameters, often involving both limbs. Finally, these outcomes were associated with reductions in neuropathic pain and gains in functional capacity and perceived QoL.

Several methodological limitations must be acknowledged. The small patient cohort and heterogeneity in STS location limited the generalizability of the findings. Furthermore, the systematic use of a unilateral crutch during all evaluation sessions, while ensuring consistency across assessments, may have influenced ambulation patterns and potentially masked residual disabilities that could emerge during free overground walking. Therefore, the results should be interpreted as reflective of crutch-assisted gait recovery rather than fully independent functional performance.

Nonetheless, these findings provided compelling evidence of the efficacy of a tailored rehabilitation protocol following radical LL-STS resection and FFMT, establishing a critical benchmark for the field. This study represented the first in-depth, multi-modal analysis of surgical and rehabilitative outcomes on gait and QoL in this patient population. The demonstrated improvements in neuromotor function, joint stability, and patient-reported outcomes highlighted the potential of personalized rehabilitation strategies. These achievements pave the way for further analysis on a larger number of patients, comparing rehabilitation pathways across different surgical treatments, the maintenance of functional recovery during long-term rehabilitative follow-up, as well as the mechanisms underlying functional recovery.

## Data Availability

All data needed to evaluate the conclusions are present in the paper. Additional data may be requested from the corresponding author upon reasonable request.

## Abbreviations

aFE: Ankle flexion/extension
ALT: Antero-Lateral Thigh
aROM: Ankle Range of Motion
BP: Bodily pain
BF: Biceps femoris
CCI: Co-Contraction Index
CF: Cognitive Function
CO: Constipation
DI: Diarrhea
DY: Dyspnea
EF: Emotional Function
EORTC QLQ-C30: European Organization for Research and Treatment of Cancer Quality-of-Life Questionnaire
FA: Fatigue
FD: Financial difficulties
FFMT: Free Functional Muscle Transfer
GH: General health
GL: Gastrocnemious Lateralis
GM: Gluteus Maximus
hFE: Hip Flexion/Extension
hROM: Hip Range of Motion
iEMG: Invasive Electromiography
IN: Insomnia
LA: Lost of Appetite
LCF-AV: Lateral circumflex femoral artery and vein
LD: Latissimus dorsi
kFE: Knee Flexion/Extension
kROM: Knee Range of Motion
L: Left
LANSS: Leeds assessment of neuropathic symptoms and signs
LL-STS: Lower limb Soft Tissue Sarcoma
M: Male
MH: Mental health
MSTS-LL: Musculoskeletal Tumor Society Rating Scale differentiated for the Lower Limb
NV: Nausea and vomiting
PA: Pain
PF: Physical function
PF-AVp: Perforating branches of the Profunda Femoris Artery
QoL: Quality of life
RE: Role emotional
RF: Rectus femoris
RFu: Role function
ROM: Range of Motion
RMS: Root Mean Square
RP: Role Physical
sEMG: Surface electromyography
SENIAM: Surface Electromyography for the Non-Invasive Assessment of Muscles
SF: Social functioning
SF-AVp: Superficial Femoral Artery and Perforating Vein
SF-36: Short Form Health Survey 36
SR: Sartorius
ST: Semitendinosus
STS: Soft Tissue Sarcoma
TA: Tibialis Anterior
TESS-LL: Toronto Extremity Salvage Score differentiated for the Lower Limb
VI: Vastus intermedius
VL: Vastus lateralis
VM: Vastus Medialis
VT: Vitality
