# Correction: Effects of a tailored rehabilitation treatment in lower limb Soft Tissue Sarcomas reconstruction: a case series

**DOI:** 10.1186/s12984-026-02019-1

**Published:** 2026-05-28

**Authors:** Andrea Demofonti, Beniamino Brunetti, Marco Germanotta, Marco Morelli Coppola, Francesca Falchini, Alice Valeri, Stefania Tenna, Sergio Valeri, Irene Giovanna Aprile

**Affiliations:** 1 IRCCS Fondazione Don Carlo Gnocchi, 50143 Florence, Italy; 2 Operative Research Unit of Plastic-Reconstructive and Aesthetic Surgery, Fondazione Policlinico Universitario Campus Bio-Medico, 00128 Rome, Italy; 3 Operative Research Unit of Plastic-Reconstructive and Aesthetic Surgery, Università Campus Bio-Medico di Roma, 00128 Rome, Italy; 4 Operative Research Unit of Soft-Tissue Sarcomas Surgery Department, Fondazione Policlinico Universitario Campus Bio-Medico, 00128 Rome, Italy

**Correction: Journal of NeuroEngineering and Rehabilitation (2026) 23:86**

10.1186/s12984-026-01878-y

In the original version of this article [1], the given and family names of Marco Morelli Coppola were incorrectly structured. The name was displayed correctly in all versions at the time of publication.

The original article has been corrected.
